# Preliminary Incidence and Trends of Infections with Pathogens Transmitted Commonly Through Food — Foodborne Diseases Active Surveillance Network, 10 U.S. Sites, 2006–2017

**DOI:** 10.15585/mmwr.mm6711a3

**Published:** 2018-03-23

**Authors:** Ellyn P. Marder, MPH, Patricia M. Griffin, Paul R. Cieslak, John Dunn, Sharon Hurd, Rachel Jervis, Sarah Lathrop, Alison Muse, Patricia Ryan, Kirk Smith, Melissa Tobin-D’Angelo, Duc J. Vugia, Kristin G. Holt, Beverly J. Wolpert, Robert Tauxe, Aimee L. Geissler

**Affiliations:** ^1^Division of Foodborne, Waterborne, and Environmental Diseases, National Center for Emerging and Zoonotic Infectious Diseases, CDC; ^2^Oregon Health Authority; ^3^Tennessee Department of Health; ^4^Connecticut Department of Public Health; ^5^Colorado Department of Public Health and Environment; ^6^University of New Mexico, Albuquerque; ^7^New York State Department of Health; ^8^Maryland Department of Health; ^9^Minnesota Department of Health; ^10^Georgia Department of Public Health; ^11^California Department of Public Health; ^12^Food Safety and Inspection Service, U.S. Department of Agriculture, Atlanta, Georgia; ^13^Center for Food Safety and Applied Nutrition, Food and Drug Administration, Silver Spring, Maryland.

Despite ongoing food safety measures in the United States, foodborne illness continues to be a substantial health burden. The 10 U.S. sites of the Foodborne Diseases Active Surveillance Network (FoodNet)* monitor cases of laboratory-diagnosed infections caused by nine pathogens transmitted commonly through food. This report summarizes preliminary 2017 data and describes changes in incidence since 2006. In 2017, FoodNet reported 24,484 infections, 5,677 hospitalizations, and 122 deaths. Compared with 2014–2016, the 2017 incidence of infections with *Campylobacter*, *Listeria*, non-O157 Shiga toxin–producing *Escherichia coli* (STEC), *Yersinia*, *Vibrio*, and *Cyclospora* increased. The increased incidences of pathogens for which testing was previously limited might have resulted from the increased use and sensitivity of culture-independent diagnostic tests (CIDTs), which can improve incidence estimates (*1*). Compared with 2006–2008, the 2017 incidence of infections with *Salmonella* serotypes Typhimurium and Heidelberg decreased, and the incidence of serotypes Javiana, Infantis, and Thompson increased. New regulatory requirements that include enhanced testing of poultry products for *Salmonella*^†^ might have contributed to the decreases. The incidence of STEC O157 infections during 2017 also decreased compared with 2006–2008, which parallels reductions in isolations from ground beef.^§^ The declines in two *Salmonella* serotypes and STEC O157 infections provide supportive evidence that targeted control measures are effective. The marked increases in infections caused by some *Salmonella* serotypes provide an opportunity to investigate food and nonfood sources of infection and to design specific interventions.

FoodNet conducts active, population-based surveillance for laboratory-diagnosed infections caused by *Campylobacter, Cryptosporidium, Cyclospora, Listeria, Salmonella,* STEC, *Shigella, Vibrio,* and *Yersinia* in 10 sites that account for approximately 15% of the U.S. population (an estimated 49 million persons in 2016). FoodNet is a collaboration among CDC, 10 state health departments, the U.S. Department of Agriculture’s Food Safety and Inspection Service (USDA-FSIS), and the Food and Drug Administration (FDA). Laboratory-diagnosed bacterial infections are defined as isolation of bacteria from a clinical specimen by culture or detection by a CIDT. CIDTs detect bacterial antigens, nucleic acid sequences, or, for STEC, Shiga toxin or Shiga toxin genes.^¶^ A CIDT-positive–only bacterial infection is a positive CIDT result without culture confirmation. *Listeria* cases are defined as isolation of *L. monocytogenes* or detection by a CIDT from a normally sterile site or from placental or fetal tissue in the instance of miscarriage or stillbirth. Laboratory-diagnosed parasitic infections are defined as detection of the parasite from a clinical specimen. Hospitalizations and deaths within 7 days of specimen collection are attributed to the infection. Surveillance for physician-diagnosed postdiarrheal hemolytic uremic syndrome (HUS) is conducted through a network of nephrologists and infection preventionists and hospital discharge data review. This report includes pediatric HUS cases identified during 2016, the most recent year for which data are available.

Incidence per 100,000 population was calculated by dividing the number of infections in 2017 by the U.S. Census estimates of the surveillance area population for 2016. Incidence measures include all laboratory-diagnosed infections reported. A negative binomial model with 95% confidence intervals (CIs) was used to estimate change in incidence during 2017 compared with that during 2014–2016 and 2006–2008. Because of large changes in testing practices since 2006, incidence comparisons with 2006–2008 used only culture-confirmed bacterial infections, and comparisons with 2014–2016 used culture-confirmed and CIDT-positive–only cases combined. For HUS, 2016 incidence was compared with that during 2013–2015.

## Cases of Infection, Incidence, and Trends

During 2017, FoodNet identified 24,484 cases of infection, 5,677 hospitalizations, and 122 deaths. The incidence of infection per 100,000 population was highest for *Campylobacter* (19.2) and *Salmonella* (16.0), followed by *Shigella* (4.3), STEC (4.2),** *Cryptosporidium* (3.7), *Yersinia* (1.0), *Vibrio* (0.7), *Listeria* (0.3), and *Cyclospora* (0.3) (Table 1). The percentage of CIDT-positive–only infections, including those that were culture-negative and those not tested by culture, were *Yersinia* (51%), *Campylobacter* (36%), *Shigella* (31%), *Vibrio* (29%), STEC (27%), *Salmonella* (9%), and *Listeria* (1%) (Figure). Compared with incidence during 2014–2016, the 2017 incidence was significantly higher for *Cyclospora* (489% increase), *Yersinia* (166% increase), *Vibrio* (54% increase), STEC (28% increase), *Listeria* (26% increase), and *Campylobacter* (10% increase) (Table 1). Bacterial infections diagnosed by CIDT increased 96% overall (range = 34%–700% per pathogen) in 2017 compared with those diagnosed during 2014–2016. Reflex culture^††^ was attempted on 71% of CIDT-positive specimens, ranging from 63% for *Campylobacter* to 100% for *Listeria* (Figure). Among specimens on which a reflex culture was performed, the percentage of positive cultures ranged from 38% for *Vibrio* to 90% for *Salmonella*.

**TABLE 1:** Incidence of bacterial and parasitic infections in 2017 and percentage change compared with 2014–2016 average annual incidence, by pathogen — FoodNet sites,* 2014–2017^†^

Pathogen	2017		2017 versus 2014–2016	
	No. of cases	Incidence rate^§^	% Change^¶^	(95% CI)
**Bacteria**				
*Campylobacter*	9,421	19.1	10	(2 to 18)
*Salmonella*	7,895	16.0	-5	(-11 to 1)
*Shigella*	2,132	4.3	-3	(-25 to 25)
Shiga toxin–producing E. coli**	2,050	4.2	28	(9 to 50)
*Yersinia*	489	1.0	166	(113 to 234)
*Vibrio*	340	0.7	54	(26 to 87)
*Listeria*	158	0.3	26	(2 to 55)
**Parasites**				
*Cryptosporidium*	1,836	3.7	10	(-16 to 42)
*Cyclospora*	163	0.3	489	(253 to 883)

**Abbreviations:** CI = confidence interval; FoodNet = CDC’s Foodborne Diseases Active Surveillance Network.

* Connecticut, Georgia, Maryland, Minnesota, New Mexico, Oregon, Tennessee, and selected counties in California, Colorado, and New York.

^†^ Data for 2017 are preliminary.

^§^ Per 100,000 population.

^¶^ Percentage change reported as increase or decrease.

** For Shiga toxin–producing *E. coli*, all serogroups were combined because it is not possible to distinguish between serogroups using culture-independent diagnostic tests. Reports that were only Shiga toxin–positive from clinical laboratories and were Shiga toxin–negative at a public health laboratory were excluded (n=518). When these were included, the incidence rate was 5.2, which was a 57% increase (CI = 33% to 85%).

**FIGURE:**
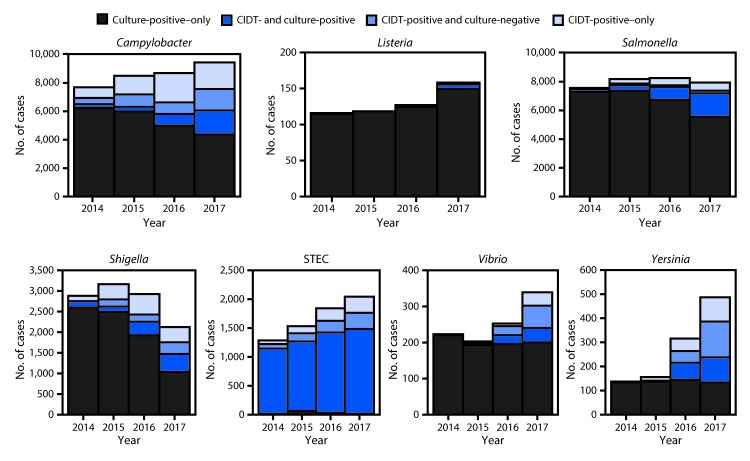
Number of infections diagnosed by culture or culture-independent diagnostic tests, by pathogen, year, and culture status — FoodNet sites,* 2014–2017†^,§^ **Abbreviations:** CIDT = culture-independent diagnostic test; FoodNet = CDC’s Foodborne Diseases Active Surveillance Network; STEC = Shiga toxin–producing *Escherichia coli*. * Connecticut, Georgia, Maryland, Minnesota, New Mexico, Oregon, Tennessee, and selected counties in California, Colorado, and New York. ^†^ Data for 2017 are preliminary. ^§^ For STEC, all serogroups were combined because it is impossible to distinguish between serogroups using CIDTs. Reports that were only Shiga toxin–positive from clinical laboratories and were Shiga toxin–negative at a public health laboratory were excluded (n=518).

Among 6,373 (89%) fully serotyped *Salmonella* isolates, the five most common were Enteritidis (incidence = 2.6 per 100,000), Typhimurium (1.4), Newport (1.3), Javiana (1.1), and the monophasic variant of Typhimurium, I 4,[5],12:i:- (0.9) (Table 2). Among the 13 most common serotypes, the incidence for Heidelberg in 2017 was 65% lower than during 2006–2008 and 38% lower than during 2014–2016 (Table 2). It was also significantly lower for Typhimurium for both periods (42% and 14%, respectively).

**TABLE 2:** Incidence of infection of the top 13 *Salmonella* serotypes in 2017 compared with 2006–2008 and 2014–2016 average annual incidence, by pathogen — FoodNet sites,* 2006–2017^†^

Serotype	2017	2017 versus 2006–2008		2017 versus 2014–2016	
	Incidence rate^§^	% Change^¶^	(95% CI)	% Change^¶^	(95% CI)
Enteritidis	2.6	3	(-11 to 20)	-8	(-21 to 7)
Typhimurium**	1.4	-42	(-48 to -34)	-14	(-24 to -2)
Newport	1.3	-5	(-22 to 16)	-19	(-34 to -2)
Javiana	1.1	99	(57 to 153)	-7	(-26 to 17)
I 4,[5],12:i:-**	0.9	35	(-5 to 74)	1	(-22 to 29)
Muenchen	0.4	-13	(-35 to 14)	-4	(-28 to 27)
Infantis	0.3	60	(19 to 113)	-20	(-39 to 6)
Montevideo	0.3	-30	(-47 to -8)	24	(-7 to 66)
Braenderup	0.3	29	(-5 to 76)	25	(-8 to 70)
Saintpaul	0.3	-36	(-53 to -14)	-20	(-40 to 9)
Thompson	0.3	70	(22 to 138)	32	(-5 to 84)
I 13,23:b:- ^††^	0.3	N/A	N/A	N/A	N/A
Heidelberg	0.2	-65	(-75 to -52)	-38	(-55 to -15)

**Abbreviations:** CI = confidence interval; FoodNet = CDC’s Foodborne Diseases Active Surveillance Network; N/A = not applicable.

* Connecticut, Georgia, Maryland, Minnesota, New Mexico, Oregon, Tennessee, and selected counties in California, Colorado, and New York.

^†^ Data for 2017 are preliminary.

^§^ Per 100,000 population.

^¶^ Percentage change reported as increase or decrease.

** Percentage change (95% CI) for Typhimurium including monophasic variant (I, 4[5],12:i:-) compared with 2006–2008 and 2014–2016 was -26% (-34% to -17%) and -11% (-20% to 0%), respectively.

^††^ Comparisons could not be calculated for serotype I 13,23,b:I because of sparse data across the entire period.

Among 1,473 STEC isolates tested for the O157 antigen, 413 (28%) were determined to be O157. Among the 766 non-O157 STEC isolates with serogroup determined, the most common were O26 (29%), O103 (26%), and O111 (18%). During 2017, the incidence of non-O157 STEC significantly increased 25% (95% CI = 9–44) compared with that during 2014–2016; incidence of STEC O157 was unchanged. However, compared with 2006–2008, the incidence of STEC O157 was significantly lower (35% decrease; 95% CI = 21–46).

FoodNet identified 57 cases of HUS in children (incidence = 0.51 per 100,000) during 2016; 35 (61%) occurred among children aged <5 years (incidence = 1.18 per 100,000). The incidence during 2016 compared with that during 2013–2015 was not significantly different among all children or those aged <5 years. The incidence among children aged <5 years significantly decreased 36% (95% CI = 8–55) in 2016 compared with 2006–2008.

## Discussion

Clinical laboratories are steadily increasing the use of CIDTs, particularly DNA-based syndrome panels, to diagnose enteric pathogens (*2*). Previously, routine stool tests typically only included methods for identifying *Salmonella*, *Campylobacter*, *Shigella*, and STEC O157 (*3*). CIDTs benefit public health by identifying illnesses caused by pathogens not captured routinely by older methods, revealing more accurate incidence estimates for some pathogens. For example, most laboratories required a specific request to test for *Cyclospora*. Because use of panel tests has risen, routine tests more often include *Cyclospora* as well as *Yersinia*, *Vibrio*, and non-O157 STEC. The increased incidence of these infections in 2017 was most likely driven by the increased use of CIDTs.

Although the number of *Salmonella* infections with CIDT-positive results increased 176% during 2017 compared with 2014–2016, the overall percentage without culture confirmation remained relatively low (9%) because of the high frequency and success of reflex culture, which is necessary for subtyping. Infections caused by serotypes Typhimurium (including I 4,[5],12:i:-) and Heidelberg have decreased considerably over the past 10 years. These declines mirror decreases in broiler chicken samples that yielded *Salmonella* and, specifically, serotype Heidelberg (USDA-FSIS, unpublished data). These declines might be partly because of industry measures to vaccinate poultry flocks against these serotypes (*4*) as well as implementation of measures by USDA-FSIS to decrease *Salmonella* in poultry and beef products.

Despite these decreases, the overall incidence of *Salmonella* has not substantially declined since 2014–2016, partly because infections with some serotypes have increased. In particular, infections caused by serotypes Javiana, Thompson, and Infantis each increased approximately 50% compared with 2006–2008. Like most serotypes, these have been linked to both food and other exposures, including animal contact (*5*). Thus, some of these infections are likely attributable to nonfood exposures. USDA-FSIS also noted an increase of >50% in the percentage of broiler chicken samples that yielded Infantis from 2006 to 2017 (USDA-FSIS, unpublished data).

The decreasing availability of STEC serogroup information, attributable to CIDTs, makes interpretation of trends difficult. However, the decreased incidence of HUS among young children during 2016 compared with that during 2006–2008 provides evidence that supports the finding of a decline in STEC O157 infections because most HUS cases are caused by STEC O157 (*6*). This decline also mirrors declines in STEC O157 in ground beef during the same period.

CIDTs pose challenges to public health when reflex culture is not performed. Without isolates, public health laboratories are unable to subtype pathogens, determine antimicrobial susceptibility, and detect outbreaks. Reflex culture recovery rates vary, which could be attributed to false positives, low numbers of bacteria, storage or transport problems, or insensitive culture techniques (*7*,*8*). Furthermore, CIDTs vary in sensitivity and specificity. Evaluations of panel tests have indicated high sensitivity and specificity, differing by test type and manufacturer. The Association of Public Health Laboratories recommends that clinical laboratories culture CIDT-positive specimens (*9*). The lack of isolates for 25% of bacterial infections in 2017 is cause for concern.

The findings in this report are subject to at least two limitations. First, the changing diagnostic landscape makes interpretation of incidence and trends difficult. In addition to actual increases in infection, increases in reported incidence might be due to some health care providers being more likely to order a CIDT because results are more quickly obtained than with traditional culture methods (*1*). Increases in incidence could also be due to increased use of DNA-based syndrome panel tests that diagnose pathogens not captured routinely by older methods. With improved sensitivity and specificity of DNA-based CIDTs, infections that previously would have remained undetected by culture methods might now be detected. Second, changes in incidence can reflect year-to-year variation rather than sustained trends.

Most foodborne illnesses can be prevented. New regulatory requirements aimed at reducing contamination of poultry meat might have contributed to decreases in incidence of infections caused by *Salmonella* serotypes Typhimurium and Heidelberg. Vaccination might also have contributed, but the extent of vaccination in poultry broiler flocks has not been reported. The declines in these and in STEC O157 infections provide supportive evidence that targeted control measures are effective. More control measures are needed and might be achieved with continued implementation of the FDA Food Safety Modernization Act,^§§^ new or revised meat and poultry performance standards, and enhanced training and guidance for industry and inspection personnel. In particular, measures targeting specific *Salmonella* serotypes, including vaccination of broiler poultry flocks, might result in a marked decrease in human illness, as has been seen in the United Kingdom (*10*).

**Summary**

**What is already known about this topic?**

The incidence of infections transmitted commonly through food has remained largely unchanged for many years. Culture-independent diagnostic tests (CIDTs) are increasingly used by clinical laboratories to detect enteric infections. CIDTs benefit public health surveillance by identifying illnesses caused by pathogens not captured routinely by previous laboratory methods.

**What is added by this report?**

Decreases in incidence of infection of Shiga toxin–producing *Escherichia coli* (STEC) O157 and *Salmonella* serotypes Typhimurium and Heidelberg have been observed over the past 10 years. These declines parallel findings of decreased *Salmonella* contamination of poultry meat and decreased STEC O157 contamination of ground beef.

**What are the implications for public health practice?**

As use of CIDTs continues to increase, higher, more accurate incidence rates might be observed. However, without isolates, public health laboratories are unable to subtype pathogens, determine antimicrobial susceptibility, and detect outbreaks. Further prevention measures are needed to decrease the incidence of infection by pathogens transmitted commonly through food.

## Footnotes

* Connecticut, Georgia, Maryland, Minnesota, New Mexico, Oregon, Tennessee, and selected counties in California, Colorado, and New York https://www.cdc.gov/foodnet.

† https://www.fsis.usda.gov/wps/portal/fsis/topics/food-safety-education/get-answers/food-safety-fact-sheets/foodborne-illness-and-disease/salmonella/sap-two-year and https://www.fsis.usda.gov/wps/wcm/connect/fb8c866a-a9b7-4b0d-81c9-0f190c4a8d4d/2011-0012F.htm?MOD=AJPERES.

§ https://www.fsis.usda.gov/wps/portal/fsis/topics/data-collection-and-reports/microbiology/ec/e-coli-o157h7-year-to-date/ecoli-o157-raw-beef-testing-data-ytd.

¶ For Shiga toxin–producing *Escherichia coli*, only CIDT reports that were positive at a state public health laboratory were counted.

** Excludes Shiga toxin-positive–only reports from clinical laboratories that were Shiga toxin–negative at a public health laboratory (n = 518).

†† Culturing of a specimen with a positive CIDT result.

§§ https://www.fda.gov/Food/GuidanceRegulation/FSMA/.
